# Editorial: Old and new psychoactive substances: Pharmacology and potential applications

**DOI:** 10.3389/fpsyt.2022.1087005

**Published:** 2023-01-04

**Authors:** Felix P. Mayer, Dino Luethi, Lorena B. Areal, Harald H. Sitte

**Affiliations:** ^1^Department of Biomedical Science, Florida Atlantic University, Jupiter, FL, United States; ^2^Division of Clinical Pharmacology and Toxicology, University Hospital Basel and University of Basel, Basel, Switzerland; ^3^Institute of Pharmacology, Center for Physiology and Pharmacology, Medical University of Vienna, Vienna, Austria; ^4^AddRess, Center for Addiction Research and Science, Medical University of Vienna, Vienna, Austria

**Keywords:** psilocybin, MDMA, ketamine, PTSD, cathinone, new psychoactive substance (NPS)

The consumption of psychoactive substances is inextricably linked with human history. Longstanding evidence supports the consumption of psychoactive substances that are found in plants and fungi (e.g., cathinone and muscimol, which are present in *Catha edulis* and *Amanita muscaria*, respectively) and alcoholic beverages. With the advent of more refined chemical approaches, isolated (e.g., cocaine) and purely synthetic [e.g., *N*-methylamphetamine and lysergic acid diethylamide (LSD)] compounds were added to the repertoire of drugs with psychoactive properties.

Various compounds that are consumed in recreational settings are also of clinical relevance, as exemplified by *d*-amphetamine and methylphenidate. Both substances are used for the treatment of attention-deficit/hyperactivity disorder (ADHD) but are also ingested for their stimulating and appetite-suppressing properties (1). Of note, the phenomenon of new psychoactive substances (NPS) that are introduced into the global and local drug markets drastically increased the number of drugs that are available for the consumer; on the flip side, these substances simultaneously increased our understanding of structure–activity relationships which could aid the design of pharmaceuticals with improved pharmacodynamics. Recently, the scientific community began to re-evaluate the therapeutic utility of a subset of recreationally consumed substances. For example, the psychedelics LSD, *N*,*N*-dimethyltryptamine (DMT), psilocybin and the dissociative anesthetic ketamine (2–4) are now being evaluated in clinical trials to assess their potential as antidepressants. The empathogen 3,4-methylenedioxymethamphetamine (MDMA) has proven effective as an adjunct to psychotherapy for posttraumatic stress disorder (PTSD) (5).

The goal of this issue was to collect manuscripts that refine and summarize our current understanding of the challenges associated with psychoactive substances as well as their molecular mechanism of action and potential clinical utility.

Di Trana et al. provide an introduction to the challenges that are associated with new synthetic opioids (NSOs) and the “opioid crisis” and comment on the emergence of synthetic benzimidazole opioids (BO) on the drug markets. The authors discuss the history of BOs and the rise of this subclass of NSOs, which presumably reflects the need for an inexpensive replacement of fentanyl analogs, also referred to as fentalogues, which dominate the European opioid market. Moreover, the authors emphasize the as of yet limited knowledge on the pharmacology of BOs and raise the concern that this class of NSOs should be monitored closely by the authorities.

Tirri et al. studied the sensorimotor responses, motor activity, and other effects in mice treated with novel *N*-methoxybenzyl derivatives of 2,5-dimethoxy-substituded phenethylamines (2C derivatives). The authors show that halogenated NBOMe derivatives are overall more effective than 2C compounds or LSD in altering visual and acoustic responses, affecting reaction time, and motor and sensory gating. These findings highlight the high potency of NBOMes and associated risks for recreational users.

The primary research article by Kiraga et al. describes the effect of psilocybin on state and trait anxiety in (self-reported) healthy volunteers when taken in a supportive group setting. When compared to baseline, the consumption of psilocybin containing truffles was found to reduce state and trait anxiety the morning after and those reductions persisted over 1 week post-ceremony. The ratings of ego dissolution and changes in neuroticism were found to be predictors of reductions in state and trait anxiety, respectively. The results presented in this article support the conclusion that the anxiolytic effects of psilocybin are associated with a fast onset and long duration in patients with sub-clinical anxiety symptoms.

The next article of this Research Topic by Huang et al. assessed the effect of psilocybin on body weight in a rodent model of obesity. Male rats were pre-conditioned with cafeteria diet until they met the criteria of obesity and then treated with either metformin or two doses of psilocybin. This original study, led by Joyce Huang, found that both metformin and psilocybin at 0.1 and 5 mg/kg (i.p.) significantly reduced body weight when compared to the control group. Considering that current pharmacological treatments for obesity are limited by their effectiveness and/or side effects, these findings could support the search for strategies for the treatment of obesity.

Ko et al. reviewed the association between the mystical experience (e.g., ego dissolution and universal interconnectedness) associated with psychedelic therapy and symptom reduction. As the authors state, they found “a significant association of correlation, mediation, and/or prediction” in 10 out of 12 studies. Yet, the authors highlight the limitations of the design and sample size of the reviewed studies and emphasize the need for randomized studies with greater sample size and diversity.

In a comprehensive review, López-Arnau et al. provide an overview on therapeutic mechanisms of MDMA, synthetic cathinones, and psychedelics. The authors furthermore discuss specific properties that may render novel stimulants and psychedelics promising as future therapeutics.

Finally, Sottile and Vida discuss the existing literature on MDMA and the potential mechanism(s) of action that underly the MDMA-mediated extinction of traumatic memories.

Overall, this collection of original articles and literature reviews provides new insights into mechanisms of action, safety, and the clinical potential of various psychoactive substances. While challenges remain, significant progress has been made in the past years regarding the characterization of NPS and the exploration of the therapeutic potential of drugs historically used for recreational purposes, which we hope was conveyed by our Research Topic. This Research Topic demonstrates the increasing interest of the scientific community in exploring the knowledge acquired by the study of structure–activity relationships and behavioral effects of recreational drugs in hopes to create novel therapeutic drugs with improved pharmacodynamics for the treatment of neuropsychiatric disease and/or to repurpose available psychoactive substances with optimized therapeutic schemes. With NPS constantly reaching the market, this is a subject that will certainly require continuous research and updates. We look forward to continued studies aiming to expand our knowledge on pharmacological mechanisms of psychoactive substances, not only for awareness of harmful effects and better management of toxicity, but also to contribute to the design of novel and improved therapeutics for neuropsychiatric disorders.

## Author contributions

FM concenptualized the Research Topic together with DL. LA and HS provided significant input.
